# Exploring In Vitro Immunomodulatory Properties of Moss *Atrichum undulatum* Extracts

**DOI:** 10.3390/plants13101349

**Published:** 2024-05-13

**Authors:** Tanja Lunić, Marija Rakić, Aneta Sabovljević, Marko Sabovljević, Tamara Filipović, Bojan Božić, Biljana Božić Nedeljković

**Affiliations:** 1 Institute of Physiology and Biochemistry “Ivan Đaja”, Faculty of Biology, University of Belgrade, 11000 Belgrade, Serbia; marija.mandic@bio.bg.ac.rs (M.R.); tasa.filipovic1@gmail.com (T.F.); bbozic@bio.bg.ac.rs (B.B.); biljana@bio.bg.ac.rs (B.B.N.); 2 Institute of Botany and Botanical Garden “Jevremovac”, Faculty of Biology, University of Belgrade, 11000 Belgrade, Serbia; aneta@bio.bg.ac.rs (A.S.); marko@bio.bg.ac.rs (M.S.); 3 Department of Botany, Institute of Biology and Ecology, Faculty of Science, Pavol Jozef Šafárik University in Košice, Mánesova 23, 04001 Košice, Slovakia

**Keywords:** axenic mosses, *Atrichum undulatum*, microglia, neuroinflammation, immunomodulation

## Abstract

Bryophytes are rich sources of diverse secondary metabolites with a wide range of biological activities, including anti-inflammatory, antitumor and antimicrobial effects. The aim of this study was to investigate the chemical composition of extracts from two different genotypes (Serbian and Hungarian) of the axenic moss *Atrichum undulatum* and evaluate the immunomodulatory potential of the prepared extracts in vitro. Both genotypes of moss samples were cultivated in vitro and subsequently extracted in a Soxhlet apparatus with methanol or ethyl acetate. The highest concentration of total phenolic compounds was found in the methanolic extract of the Serbian genotype (54.25 mg GAE/g extract), while the ethyl acetate extract of the Hungarian genotype showed the highest concentration of phenolic acids (163.20 mg CAE/extract), flavonoids (35.57 mg QE/extract), and flavonols (2.25 mg QE/extract). The extracts showed anti-neuroinflammatory properties by reducing the production of reactive oxygen species, nitric oxide, and tumor necrosis factor alpha by lipopolysaccharide-stimulated microglial cells. Moreover, they mitigated the cytotoxic effects of the pro-inflammatory mediators produced by activated microglia on neurons. The data obtained suggest that extracts from *A. undulatum* moss have promising anti-neuroinflammatory and neuroprotective properties, making them interesting candidates for further research to combat neuroinflammation.

## 1. Introduction

Bryophytes, a diverse group of plants, occupy a unique place in the botanical world due to their dominant haploid gametophyte life cycle phase [1]. This feature distinguishes them from higher plants and makes them particularly interesting for the study of plant evolution and reproductive strategies. Bryophytes can be broadly divided into three main lineages, namely, mosses (Bryopsida), hornworts (Anthocerotopsida), and liverworts (Marchantiopsida), with species varying in size from a few millimeters to over 70 cm [2]. They thrive in moist habitats such as rocks, stones, and tree bark and can be found on every continent, with the exception of marine areas. With more than 14,000 identified species [3], mosses contribute significantly to the world’s plant diversity. Bryophytes have a rich history in traditional medicine, with documented use in China, India, and among indigenous populations for the treatment of various ailments, including ulcers, burns, eczema, and wounds. These ancient remedies highlight the potential therapeutic value of bryophytes [4,5,6]. Nevertheless, these plants have often been overlooked in biomedical research due to their small size and limited biomass (especially clean, i.e., axenic material).

Bryophytes are known to be a source of diverse secondary metabolites, encompassing glycosides, fatty acid derivatives, phenols, and terpenoids [7]. These compounds provide tolerance to environmental stresses such as UV radiation, drought, and extreme temperatures. Additionally, they serve as carriers of various biological activities, such as antibacterial, antifungal, antitumor, anti-inflammatory, and anti-neurodegenerative ones [7,8]. One notable moss species, acrocarpous *Atrichum undulatum* (Hedw.) P. Beauv., belongs to the class Bryopsida and is one of the largest terrestrial mosses in Europe. *Atrichum undulatum* is characterized by a rather narrow costa with filaments along it, transversely wavy phyloids, and resistance to drought and metal pollution, and it generally represents a resilient and adaptable species [9,10,11,12]. Although this moss is commonly found in Europe [13], there is limited research on the chemical composition and biological activities of *A. undulatum* [14,15]. Typically, biological assays focus on analyzing its potential antioxidant, antimicrobial, and antiproliferative properties [1,9,16,17].

Inflammation is an essential mechanism of the body’s innate immune response and plays an important role in defending against infection, repairing tissue damage, and maintaining overall homeostasis in the body. Inflammation can be acute or chronic, with acute inflammation being a rapid response involving various immune cells and signaling molecules. Chronic inflammation, which can result from an unresolved infection or autoantigens, can lead to tissue damage [18]. In some cases, peripheral inflammation, leads to the neuroinflammation in the central nervous system (CNS). Neuroinflammation involves the activation of microglia, the immune cells residing in the CNS, and is associated with neurodegenerative diseases such as Alzheimer’s and Parkinson’s disease [19,20]. Microglial cells play a critical role in CNS health by influencing neuron survival, synaptogenesis, and tissue homeostasis. Under pathological conditions, such as the presence of lipopolysaccharides (LPSs), microglial cells become activated, alter their morphology, and produce pro-inflammatory cytokines (interleukin 1, IL-1; interleukin 6, IL-6; tumor necrosis factor alpha, TNF-α), reactive oxygen species (ROS), nitric oxide (NO), and other inflammatory mediators that further contribute to neuroinflammation [21,22]. Understanding inflammation and its consequences, including neuroinflammation, oxidative stress, and the production of pro-inflammatory molecules, in the context of various pathological conditions, especially neurodegenerative diseases, is crucial. Therefore, the aim of this study was to investigate the immunomodulatory potential of two different genotypes of *A. undulatum* moss extracts prepared using methanol or ethyl acetate solvents by evaluating their chemical composition, biocompatibility, and effect on neuroinflammation, using the in vitro model of neuroinflammation mediated by microglia.

## 2. Results

### 2.1. Extraction Yield

The calculation of moss extraction yield was achieved by considering both the mass of dry moss used in the extraction process and the mass of the resulting dry extract. Based on the findings presented in Table 1 regarding the extraction yields, it can be observed that the extracts E2 and E4 exhibited the highest yields, 5.30 and 4.21%, respectively. These results demonstrate that the extraction of moss *A. undulatum* from both Serbian and Hungarian genotypes using ethyl acetate resulted in higher yields when compared to methanol-based extraction.

### 2.2. Chemical Characterization of Extracts

The results of total phenolic content (TPC), total phenolic acid content (TPAC), total flavonoid content (TFC), and total flavonol content (TFlC) are presented in Table 2.

The results shown in Table 3 indicate the presence of phenolic compounds in all examined extracts. By comparing the TPC among the extracts, it is evident that the E1 extract exhibits the highest concentration of total phenolic compounds, 54.25 mg GAE/g extract. Furthermore, when comparing different extracts from the Serbian genotype (E1 and E2), it can be concluded that the concentration of phenols is notably higher in the extracts prepared using methanol, while, in the case of extracts from the Hungarian genotype (E3 and E4), there is an increase in the phenolic concentration observed in the extracts prepared using ethyl acetate. Regarding TPAC, extract E4 exhibited the highest concentration of phenolic acids (163.20 mg CAE/g extract) when comparing the concentrations across the examined extracts. A comparison between the concentrations of phenolic acids in the extracts derived from Serbian and Hungarian moss genotypes indicates that the ethyl acetate extracts (E2 and E4) yielded a higher phenolic acid content compared to the methanol extracts (E1 and E3). Flavonoids were also present in all the tested extracts. Specifically, the concentrations of flavonoids in the E2, E3, and E4 extracts were notably higher than those in the E1 extract, with the E4 extract exhibiting the highest concentration among them, 35.57 mg QE/g extract. Flavonols were detected in the E2, E3, and E4 extracts, with the E4 extract displaying the highest TFlC, 2.25 mg QE/g extract.

### 2.3. Biocompatibility of Extracts

**Figure 1:**
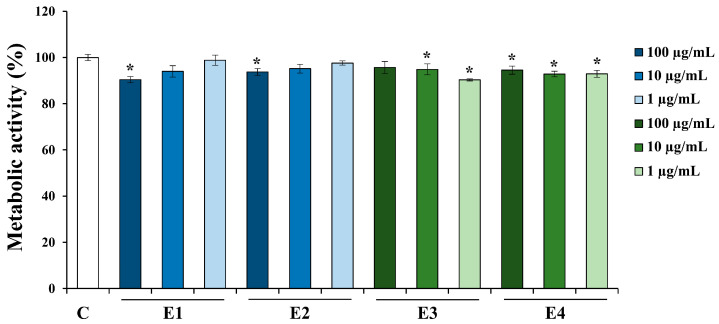
Biocompatibility testing of *Atrichum undulatum* moss extracts. C—control without extract; E1—methanol as a solvent, Serbian moss genotype; E2—ethyl acetate as a solvent, Serbian moss genotype; E3—methanol as a solvent, Hungarian moss genotype; and E4—ethyl acetate as a solvent, Hungarian moss genotype. * *p* < 0.05 vs. C.

The biocompatibility of the *A. undulatum* moss extracts was assessed using the L929 mouse fibroblast cell line. The cells were cultured and treated with the extracts, followed by conducting an MTT assay to assess metabolic activity. The results of the MTT assay are represented as a percentage of metabolic activity relative to the untreated control cells (C), which have been assigned a value of 100%. These results are depicted in Figure 1.

The obtained results reveal that the metabolic activity of the cells treated with the investigated extracts, in all the tested concentrations, exceeds 90%. These findings strongly suggest that the investigated *A. undulatum* moss extracts do not exhibit cytotoxic effects, meaning that they do not affect the viability of L929 cells. This makes them biocompatible and well-suited for further in vitro investigations.

### 2.4. The Influence of the Extracts on the Functional Characteristics of Microglia

**Figure 2:**
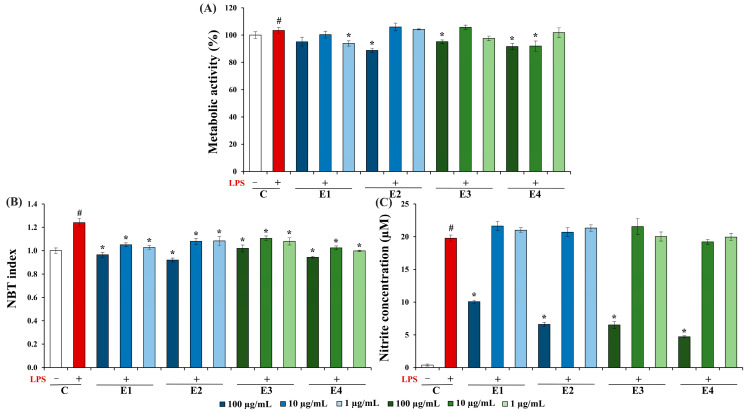
Effects of *Atrichum undulatum* moss extracts on (**A**) metabolic activity of BV-2 cells; (**B**) ROS production by BV-2 cells; and (**C**) NO production (evaluated by nitrite concentration) by BV-2 cells. C—control without (C−) or with LPS (C+); E1—methanol as a solvent, Serbian moss genotype; E2—ethyl acetate as a solvent, Serbian moss genotype; E3—methanol as a solvent, Hungarian moss genotype; and E4—ethyl acetate as a solvent, Hungarian moss genotype. # *p* < 0.05 vs. C−; * *p* < 0.05 vs. C+.

**Figure 3:**
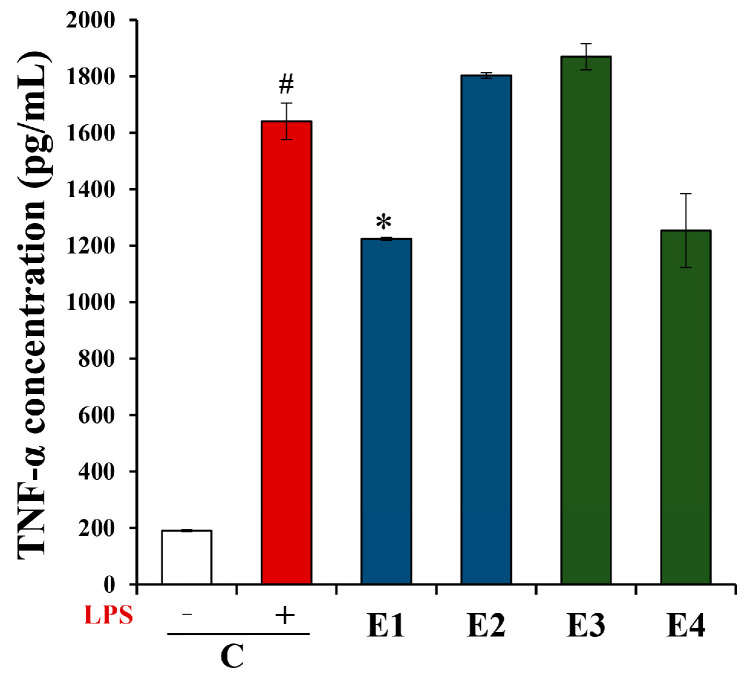
Effects of *Atrichum undulatum* moss extracts on TNF-α production by BV-2 cells. C—control without (C−) or with LPS (C+); E1—methanol as a solvent, Serbian moss genotype; E2—ethyl acetate as a solvent, Serbian moss genotype; E3—methanol as a solvent, Hungarian moss genotype; E4—ethyl acetate as a solvent, Hungarian moss genotype. # *p* < 0.05 vs. C−; * *p* < 0.05 vs. C+.

An MTT assay was employed to investigate the impact of the *A. undulatum* moss extracts on the metabolic activity of un- and LPS-stimulated BV-2 microglia cells (Figure 2A). The influence of the *A. undulatum* moss extracts on ROS production (Figure 2B), NO production (Figure 2C), and TNF-α production (Figure 3) by LPS-stimulated BV-2 microglia cells was evaluated using the NBT assay, the Griess assay, and ELISA, respectively. LPS was utilized as the stimulating agent for one subset of extract-untreated cells (C+) and for all the cells subjected to the extract treatments (E1–4+).

The results obtained (Figure 2A) reveal that the stimulation of cells with LPS resulted in increased metabolic activity (103.5%) compared to the unstimulated control cells (100%). When we compare the metabolic activity of the cells stimulated with LPS and treated with extracts to those stimulated with LPS alone, it is evident that the metabolic activity of the cells treated with various concentrations of extracts consistently exceeded 88%. These results suggest that the tested *A. undulatum* moss extracts do not exhibit any toxic effects, meaning that they do not compromise the viability of LPS-activated BV-2 microglia cells. Consequently, these extracts exhibit biocompatibility tested on microglia and are well-suited for further in vitro testing.

The influence of *A. undulatum* moss extracts on ROS production by LPS-stimulated BV-2 microglia cells was evaluated using the NBT test. The results of this experiment are presented in Figure 2B as the NBT index in a comparison with control cells, assigned a value of 1. The findings presented in Figure 2B reveal a significant increase in the production of ROS by BV-2 microglia cells when exposed to LPS (NBT index = 2.4). When comparing extract-treated LPS-stimulated cells with the LPS-stimulated control (C+), it becomes evident that treatment with all the investigated extracts leads to a statistically significant reduction in the production of ROS by moss-treated cells. These results provide compelling evidence of the anti-neuroinflammatory potential of the investigated *A. undulatum* moss extracts.

The Griess reaction was employed for assessing the impact of *A. undulatum* moss extracts on NO production by LPS-stimulated BV-2 microglia cells evaluated by the nitrite concentration in the supernatants. The results are shown in Figure 2C, representing nitrite concentration in µM. The results obtained show a significant elevation in NO production by BV-2 microglia cells when stimulated with LPS (C+), from 0.38 to 19.78 µM. When comparing LPS-stimulated cells and cells treated with moss extracts, a statistically significant reduction in NO production can be observed, although only at the highest applied concentrations of extracts (100 µg/mL). These findings confirm the anti-neuroinflammatory potential of the *A. undulatum* moss extracts that were examined in this research. Additionally, extracts from moss *A. undulatum* at a final concentration 100 µg/mL were further evaluated for their effects on the production of pro-inflammatory cytokine TNF-α by LPS-stimulated BV-2 microglial cells, and the results are presented in Figure 3.

The results presented in Figure 3 demonstrate that BV-2 microglia cells exhibit increased production of TNF-α when stimulated with LPS (1607 pg/mL), compared to unstimulated cells (190.3 pg/mL). Upon comparing the cells treated with extracts to the LPS-stimulated control (C+), it is evident that the E1 extract significantly decreases TNF-α production by these cells (1219.5 pg/mL). This highlights the potential of the methanolic extract from the Serbian genotype of *A. undulatum* moss to mitigate the production of the pro-inflammatory cytokine TNF-α, indicating its anti-neuroinflammatory properties.

### 2.5. The Influence of the Extracts on the Viability of Neurons

**Figure 4:**
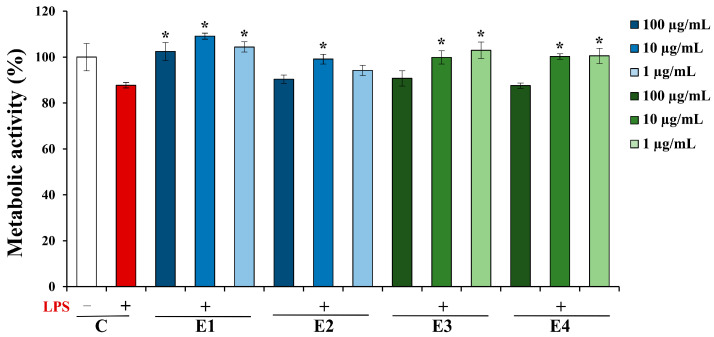
Effect of *Atrichum undulatum* moss extracts on the metabolic activity of SH-SY5Y cells. C—control without (C−) or with LPS (C+); E1—methanol as a solvent, Serbian moss genotype; E2—ethyl acetate as a solvent, Serbian moss genotype; E3—methanol as a solvent, Hungarian moss genotype; and E4—ethyl acetate as a solvent, Hungarian moss genotype. * *p* < 0.05 vs. C+.

An MTT assay was also employed to investigate the impact of *A. undulatum* moss extracts on the metabolic activity of SH-SY5Y neurons treated with supernatants from BV-2 microglia cells previously stimulated with LPS (Figure 4).

The results presented in Figure 4 show that the supernatants of BV-2 microglial cells previously stimulated with LPS (labeled C+) do not lead to any significant change in the metabolic activity of SH-SY5Y neurons (87.8% for C+ vs. 100% for C−). When comparing the effect of the supernatants of LPS-stimulated BV-2 cells treated with extracts to the LPS-stimulated control (C+), it can be seen that the majority of the extracts tested exerts a positive effect on the LPS-stimulated BV-2 cells and their supernatants normalize the metabolic activity of SH-SY5Y neurons, bringing them to a level equivalent to that of the untreated control cells (C−). It is noteworthy that E1 shows a statistically significant neuroprotective effect at all the applied concentrations; E2 shows this effect at a concentration of 10 µg/mL, while extracts E3 and E4 show the highest neuroprotective effect at concentrations of 10 and 1 µg/mL, respectively.

## 3. Discussion

The present study focused on the chemical characterization and immunomodulatory activity determination of axenic moss *A. undulatum* extracts, prepared using methanol and ethyl acetate as solvents. The chemical analysis revealed the presence of various phenolic compounds, with the Serbian genotype moss extract prepared with methanol showing the highest total phenol content, while the Hungarian genotype moss extract obtained with ethyl acetate proved to be rich in phenolic acids, flavonoids, and flavonols. This aligns with prior research, indicating that ethyl acetate is an effective solvent for extracting phenolic acids, flavonoids, and flavonols in mosses [23]. The chemical composition of moss *A. undulatum* was investigated earlier by analyzing fatty acid composition of chloroform/methanol moss extracts. In that study, eight fatty acids were identified in extracts of this species, including linoleic acid, palmitic acid, α-linolenic acid, oleic acid, arachidonic acid, stearic acid, cis-5,8,11,14,17-eicosapentaenoic acid, and behenic acid [24]. Moreover, fatty acids such as palmitoleic acid, n-hexadecanoic acid, eicosanoic acid, caproic acid, erucic acid, and others have also been detected in butanol extracts of *A. undulatum* [16]. In addition to exploring fatty acids as secondary metabolites, several studies have examined the total phenolic content of extracts derived from *A. undulatum* moss. As it is shown, the total content of phenols in the ethanolic extract of *A. undulatum* collected in the Czech Republic was estimated to be 8.72 ± 0.80 mg GAE/g dry extract [1]. Another study investigated the total phenolic content in the moss cultivated under different light and dark conditions (16/8 vs. 8/16 day length) and discovered that moss grown under conditions of a longer day length (16 h out of 24 h) has a higher phenolic content than plants grown under longer dark (16 h out of 24 h) conditions [25].

In the present study, the total phenolic content of axenic moss extracts *A. undulatum* varied from 27.21 ± 0.81 mg GAE/g dry extract in the methanol extract of the Hungarian moss genotype to 54.25 ± 1.07 mg GAE/g dry extract in the methanol extract of the Serbian moss genotype. These results indicate a higher content of total phenols in the extracts of axenic moss *A. undulatum* obtained using methanol and ethyl acetate, in comparison to the studies mentioned previously [1,25]. The observed variations may be attributed to differences in extraction solvents, extraction techniques, as well as the fact that the moss used in the present study was grown under controlled and non-variable in vitro conditions, ultimately leading to a higher phenolic content in the investigated extracts. While the fatty acid and total phenolic content of moss *A. undulatum* have been characterized in earlier-mentioned studies, to the best of our knowledge, the present study is the first to investigate the total content of phenolic acids, flavonoids, and flavonols in this moss species. Given that the compounds identified in *A. undulatum* moss extracts display diverse biological activities and hold therapeutic potential for various human pathological conditions, the subsequent phase of this study aimed to evaluate the immunomodulatory properties of these extracts. The focus was specifically on their potential in alleviating neuroinflammation, a crucial aspect of numerous neurodegenerative disorders.

Neurodegenerative diseases represent a significant global health concern, characterized by neuroinflammation and the gradual loss of neurons. The excessive activation of microglia cells in the CNS plays a crucial role in chronic inflammation and the pathology associated with these diseases [26,27]. Namely, it has been shown that oxidative damage to neurons is caused by the action of pro-inflammatory mediators produced by microglia cells, including ROS, NO, as well as pro-inflammatory cytokines, such as TNF-α [28]. Hence, the regulation of microglial activity can play a crucial role in maintaining the proper functioning of neurons and preserving CNS homeostasis. In the present study, the anti-neuroinflammatory potential of *A. undulatum* moss extracts was revealed. The most pronounced effect was observed with methanolic extract from the Serbian genotype of *A. undulatum*, showing a statistically significant reduction in the production of ROS, NO, and TNF-α by LPS-stimulated BV-2 microglia cells. The previous literature has indicated similar anti-inflammatory and anti-neurodegenerative activities in extracts from other moss species like *H. cupressiforme* and *Hedwigia ciliata* [23,29,30]. Additionally, another study demonstrated that water extracts from peat moss effectively reduced LPS-stimulated gene expression and the production of NO, TNF-α, and IL-1β in RAW 264.7 macrophages [31]. This inhibitory effect involved the downregulation of NF-κB phosphorylation and the inactivation of p38 MAPK and JNK in LPS-stimulated RAW 264.7 cells [31]. As for moss *A. undulatum*, to the best of our knowledge, the present study represents the first examination of its extracts’ potential for combating neuroinflammation in LPS-stimulated BV-2 microglia cells.

It is important to highlight that the methanol extract, which exhibited the most promising anti-inflammatory results, also contained the highest levels of total phenols known for their anti-inflammatory properties. This observation is consistent with previous research demonstrating a correlation between the consumption of phenolic-rich foods and a reduction in the inflammatory response. While the exact mechanisms behind the anti-inflammatory activity of phenolic compounds remain under investigation, various groups of phenolic compounds have demonstrated inhibitory effects on inflammatory mediators, including the regulation of NF-κB activation, iNOS expression, NO, and cytokine production [32,33].

Finally, when assessing the neuroprotective effects of the extracts, it was observed that all the tested *A. undulatum* moss extracts recovered the metabolic activity of SH-SY5Y neurons, bringing them to the levels of the control cells. This highlights the neuroprotective potential of the investigated *A. undulatum* moss extracts, potentially mediated by their previously demonstrated anti-neuroinflammatory effects. These results align with observations from studies on *H. cupressiforme* moss extracts, demonstrating neuroprotective effects through the reduction in neurotoxicity mediated by cytotoxic factors released from LPS-stimulated BV-2 microglia cells on SH-SY5Y neurons [29]. However, in the case of moss *A. undulatum*, this study represents the first exploration into the potential neuroprotective effects of its extracts on SH-SY5Y neurons.

## 4. Materials and Methods

### 4.1. Chemicals

The complete cell culture medium used for cell-related experiments comprised RPMI 1640 medium supplemented with 10% inactivated Fetal Bovine Serum (FBS), glucose, and 1% antibiotics (penicillin/streptomycin), all sourced from Sigma-Aldrich (St. Louis, MO, USA). Dimethyl sulfoxide, lipopolysaccharide from *Escherichia coli* O111:B4 (LPS) for cell stimulation, 3-(4,5-dimethylthiazole-2-yl)-2,5-diphenyltetrazolium bromide (MTT) for determining metabolic activity, nitroblue tetrazolium chloride (NBT) for assessing ROS production, and Griess reagent for assessing NO production were obtained from Sigma-Aldrich (St. Louis, MO, USA). MTT and NBT were dissolved in sterile PBS at a concentration of 5 mg/mL.

### 4.2. Plant Material

Two distinct geotypes of *A. undulatum*, originating from Serbia and Hungary, were cultivated in axenic culture following previously established methods [34], optimized for laboratory conditions. These geotypes, genetically distinguished and considered genotypes [13], have vouchers maintained in BEOU-Bryo collections. The Serbian material, sourced from Kosmaj Mt in Central Serbia (BEOU-Bryo 01832, collected and identified by Aneta Sabovljevic and Marko Sabovljevic on 18 April 2009), and the Hungarian material, collected from Lillafüred, Bükk National Park in North Hungary (BEOU-Bryo 04612, collected and identified by Marko Sabovljevic on 24 April 2012), were initially preserved in an herbarium before utilizing their dried spores to establish new axenic lines in vitro.

The mosses were cultivated at 18 ± 2 °C under cool-white, fluorescent light (47 mmol m^−2^ s^−1^), with a 16/8 h day/night cycle. They were cultured on MS half-strength basic medium [35] and transferred to a fresh medium every 6 weeks until reaching the desired biomass. The harvested biomasses were stored at −70 °C prior to the extraction procedure.

### 4.3. Extraction

Following collection from in vitro culture, the moss materials originating from Serbian and Hungarian genotypes were subsequently dried and pulverized in preparation for extraction. For the extraction procedure, 1 g of moss plant material from each genotype was weighed, and 15 mL of an appropriate solvent, either methanol or ethyl acetate, was added. By using a Soxhlet apparatus, the extraction was conducted for a duration of 10 h, which was determined based on previously established literature data indicating this timeframe as being optimal for efficient extraction [23,30]. The extracts were subjected to solvent evaporation to the constant mass by a vacuum evaporator and then stored in a light-protected and refrigerated environment at 4 °C. Depending on the solvent type and the genotype of the moss *A. undulatum* used for the extraction, the extracts were labeled as E1, E2, E3, and E4 (Table 3).

### 4.4. Chemical Characterization of A. undulatum Moss Extracts

Dry moss *A. undulatum* was used to prepare extracts at a concentration of 1 mg/mL in an appropriate solvent. The extracts prepared in this way were used to determine the total content of phenols, phenolic acids, flavonoids, and flavonols.

#### 4.4.1. Determination of Total Phenolic Content

The determination of the total phenolic content followed the protocol established by the Folin–Ciocalteu method [36], as previously described [23]. To prepare a calibration curve, gallic acid standards were dissolved in distilled water, spanning concentrations from 0.005 to 0.200 mg/mL. Following the preparation and incubation of all the samples for a period of 120 min at room temperature, the absorbance of the solutions was measured using a Microtiter plate reader (Multiscan Sky Thermo Scientific, Vantaa, Finland) at a wavelength of 740 nm. The total phenolic content in the samples was subsequently calculated employing the calibration curve equation and expressed in terms of gallic acid equivalents (GAEs) as milligrams of GAE per gram of dry extract (mg GAE/g dry extract).

#### 4.4.2. Determination of Total Phenolic Acid Content

The determination of the total phenolic acid content was conducted in accordance with the methodology previously described [37], with certain modifications [23]. For the construction of a calibration curve, caffeic acid dissolved in 50% ethanol at concentrations ranging from 0.0078 to 1 mg/mL was employed as the standard. The absorbance of the solutions was measured without any incubation period, using a Microtiter plate reader (Multiscan Sky Thermo Scientific, Finland) at a wavelength of 490 nm. The total phenolic acid content in the samples was determined by applying the calibration curve equation and expressed as caffeic acid equivalents (CAEs) in milligrams of CAE per gram of dry extract (mg CAE/g dry extract).

#### 4.4.3. Determination of Total Flavonoid Content

The determination of the total flavonoid content was conducted following the procedure by Park et al. [38], as previously described [23]. For the creation of a calibration curve, quercetin served as the standard and was dissolved in 96% ethanol at concentrations ranging from 0.005 to 0.200 mg/mL. Following incubation for a period of 40 min at room temperature, the absorbance was measured at 415 nm using a Microtiter plate reader (Multiscan Sky Thermo Scientific, Finland). The total flavonoid content in the samples was determined using the calibration curve equation and expressed in quercetin equivalents (QEs) as milligrams of QE per gram of dry extract (mg QE/g dry extract).

#### 4.4.4. Determination of Total Flavonol Content

The determination of the total flavonol content was carried out following the established protocol [37] with certain adaptations, as previously described [23]. For the construction of a calibration curve, quercetin dissolved in 100% methanol was utilized at concentrations ranging from 0.0078 to 1 mg/mL. Following an incubation period of 150 min at room temperature, the absorbance was measured at 440 nm using a Microtiter plate reader (Multiscan Sky Thermo Scientific, Finland). The total flavonol content in the samples was calculated based on the calibration curve equation and expressed in quercetin equivalents (QEs) as milligrams of QE per gram of dry extract (mg QE/g dry extract).

### 4.5. Cultivation of Cells

Three distinct cell lines were employed to assess the immunomodulatory effects of the *A. undulatum* moss extract: L929 fibroblasts, BV-2 microglia cells, and SH-SY5Y neuron cell lines. All the cells were obtained from the American Tissue Culture Collection (ATCC, Manassas, VA, USA). The cell lines were cultivated in sterile plastic flasks as monolayers, using the RPMI-1640 medium. Cultivation took place at a temperature of 37 °C within a humidified environment containing 5% CO_2_. A subculture was completed 2–3 times per week upon reaching a confluency level of ≥70%, which was confirmed through microscopic examination.

### 4.6. Treatment of Cells

Prior to any treatment, 10,000 cells per well were seeded for the L929 and BV-2 cell line, while 20,000 cells per well were seeded for the SH-SY5Y cell line (100 µL). Following seeding, the plates were subjected to an incubation period of 24 h. Stock solutions of the extracts were prepared in dimethyl sulfoxide (DMSO). The extracts intended for the cell treatment were further diluted in a cell culture medium to achieve final concentrations of 100, 10, and 1 µg/mL in each well of the culture plate.

Following a 24 h cell incubation period, 100 µL of medium containing the investigated moss extract was added to each well of the microplate. Untreated cells served as the controls. For the BV-2 cells, upon reaching confluency, LPS was introduced at a concentration determined in preliminary experiments, specifically 10 µg/mL of culture. Following an additional 24 h incubation, multiple assays were performed, including the assessments of metabolic activity, as well as the production of ROS, NO, and TNF-α by the cells. For neurons, after 24 h of incubation, supernatants (100 μL) from LPS-stimulated BV2 cells treated with moss extracts were added to a 96-well microplate seeded with SH-SY5Y neurons. Incubation was continued for another 24 h. The metabolic activity of the SH-SY5Y cells was subsequently measured using the MTT assay.

### 4.7. Determination of Metabolic Activity of Cells

The effects of the respective extracts on the cells’ metabolic activity were investigated by an MTT assay [39], as previously described [23]. The absorbance of reduced MTT was assessed by measuring the absorbance of the solution at 540/670 nm. The results are reported as a percentage of metabolic activity relative to the control, which was assigned a value of 100%.

### 4.8. Determination of Reactive Oxygen Species Production by Cells

The effects of the respective extracts on ROS production by the cells were determined by an NBT assay [40], as previously described [23]. The degree of reduction in the NBT was assessed by measuring the absorbance of the solution at 540/670 nm. The results are reported as the NBT index relative to the control, with the control being assigned a value of 1.

### 4.9. Determination of Nitric Oxide Production by Cells

The effects of the respective extracts on NO production by the cells were determined by a Griess assay [41], as previously described [23]. The intensity of the red diazo dye was quantified via the spectrophotometric measurement of the absorbance at 540 nm. The intensity of the red color is directly proportional to the concentration of nitrite and, indirectly, to the concentration of NO produced by BV-2 microglia cells.

### 4.10. Determination of Tumor Necrosis Factor Alpha Production by Cells

Supernatants of LPS-stimulated BV-2 cells from the experiments above were collected, and the concentration of cytokine TNF-α was determined. The quantification of cytokine was carried out with an ELISA kit according to the manufacturer’s suggestions (R&D Systems, Minneapolis, MN, USA). The results are expressed in pg/mL.

### 4.11. Microglia Supernatant Transfer Model to SH-SY5Y Neurons

A microglia supernatant transfer model was employed to investigate the potential impact of the *A. undulatum* moss extract on the production of soluble molecules by LPS-stimulated BV-2 microglia cells, which, in turn, exerted a cytotoxic effect on SH-SY5Y neurons. Subsequently, after SH-SY5Y neurons were treated with 100 μL of the supernatants derived from stimulated BV-2 cells treated with extracts for 24 h, an MTT test [39] was conducted to assess the metabolic activity of the neurons.

### 4.12. Statistical Analysis

Statistical processing of the results was carried out in the SPSS (Statistical Package for the Social Sciences, IBM SPSS Statistics for Windows, Version 25.0., IBM Corporation, Armonk, NY, USA) program. The cells were plated in quadruplicate for all the assays. Within the SPSS program, all the numerical parameters were presented as the mean value of the measurement ± standard error. The minimum probability value taken as statistically significant was *p* < 0.05.

## 5. Conclusions

In summary, this study highlights the therapeutic potential of *A. undulatum* moss extracts, shedding light on their secondary metabolite content, anti-neuroinflammatory properties, and neuroprotective effects. Among all the examined extracts, the methanolic extract from the Serbian genotype of *A. undulatum* moss emerged as the most effective. These findings not only underscore the importance of mosses in medicinal contexts but also offer promising avenues for the development of novel treatments for neuroinflammatory and neurodegenerative diseases. It is crucial to highlight the importance of cultivating mosses in a controlled setting, as it enables the generation of substantial plant quantities with uniform properties, a critical factor given the inherent small size of mosses. The controlled growth of plant material reduces the pressure on native population and contributes to population stability and conservation efforts. The axenically grown material can, thus, be adjusted by genotype selection or the subject of elicitation by external factors as a source of raw target materials/compounds in the desired quality and quantity.

## Figures and Tables

**Table 1:** Extraction yield for *Atrichum undulatum* extracts.

Label	Dry Extract (g)	Dry Moss (g)	Yield (%)
E1	0.0160	1	1.60
E2	0.0530	1	5.30
E3	0.0119	1	1.19
E4	0.0421	1	4.21

E1—methanol as a solvent, Serbian moss genotype; E2—ethyl acetate as a solvent, Serbian moss genotype; E3—methanol as a solvent, Hungarian moss genotype; and E4—ethyl acetate as a solvent, Hungarian moss genotype.

**Table 2:** Chemical characterization of moss *Atrichum undulatum* extracts.

Samples	TPC (mg GAE/ g Extract)	TPAC (mg CAE/ g Extract)	TFC (mg QE/ g Extract)	TFlC (mg QE/ g Extract)
E1	54.25 ± 1.07	45.13 ± 3.30	2.75 ± 0.39	ND
E2	35.08 ± 0.57	129.96 ± 1.84	28.03 ± 1.09	2.16 ± 0.36
E3	27.21 ± 0.81	65.69 ± 2.43	28.79 ± 0.86	0.75 ± 0.47
E4	35.29 ± 0.58	163.20 ± 3.88	35.57 ± 1.42	2.25 ± 0.48

E1—methanol as a solvent, Serbian moss genotype; E2—ethyl acetate as a solvent, Serbian moss genotype; E3—methanol as a solvent, Hungarian moss genotype; E4—ethyl acetate as a solvent, Hungarian moss genotype; and ND—not detected.

**Table 3:** Extract labels, solvents, and genotypes of moss *Atrichum undulatum* used in this research.

Label	Solvent	Genotype
E1	methanol	Serbian
E2	ethyl acetate	Serbian
E3	methanol	Hungarian
E4	ethyl acetate	Hungarian

## Data Availability

Data are contained within the article.
